# Living with Companion Animals, Physical Activity and Mortality in a U.S. National Cohort

**DOI:** 10.3390/ijerph7062452

**Published:** 2010-05-28

**Authors:** Richard F. Gillum, Thomas O. Obisesan

**Affiliations:** Department of Medicine, College of Medicine, Howard University, 2041 Georgia Avenue, Washington, DC, 20060, USA; E-Mail: tobisesan@howard.edu

**Keywords:** domestic animals, physical activity, mortality, survival

## Abstract

Living with a canine companion is postulated to increase physical activity. We test the hypotheses that adults living with a canine companion have a higher level of physical activity and reduced mortality risk compared to those not living with a companion animal. A U.S. national health survey with longitudinal mortality follow-up studied 11,394 American men and women aged 40 years and over examined in 1988–1994 followed an average 8.5 years. Measurements at baseline included self-reported companion animals in the household, socio-demographics, health status, physical and biochemical measurements. Outcome measures were leisure-time physical activity (LTPA), and death from all causes. Death during follow-up occurred in 3,187 persons. In bivariate cross-sectional analyses living with a dog was associated with more frequent LTPA and higher survival. In proportional hazards regression analysis, no significant interaction of age, gender or ethnicity with animals was found. After adjusting for confounding by baseline socio-demographics and health status at ages 40+, the hazards ratio (95% confidence limits) for living with a canine companion compared to no animals was 1.21(1.04–1.41, p < 0.001). After also controlling for health behaviors, blood pressure and body mass, C-reactive protein and HDL-cholesterol, the HR was 1.19 (0.97–1.47, NS). In a nationwide cohort of American adults, analyses demonstrated no lower risk of death independent of confounders among those living with canine or feline companions, despite positive association of canine companions with LTPA.

## 1. Introduction

Living in a home with a dog or other animal is highly prevalent in the U.S. and U.K. The 2005–2006 National Pet Owners Survey showed that pet ownership was at its highest level, with 63 percent of all U.S. households owning a pet which equates to more than 69 million households, up from 64 million in 2002 and 51 million in 1988 when tracking began [1]. Two studies have been published suggesting that dog owners had lower mortality risks following acute myocardial infarction, but the number of deaths was small, data on physical activity were unavailable and the studies were not population-based [2,3]. Similarly, a study in a self-selected Australian sample suggested a more favorable standard cardiovascular risk factor profile and a US/Canadian report suggested more favorable heart rate variability in pet owners than others [4,5]. However, two studies of population-based Australian samples failed to replicate the former finding or demonstrate effects on self-reported physical or mental health [6–8]. Few population-based studies have assessed the impact of dog ownership on owners’ physical activity, although existing data suggest an increase [9]. Although the significant associations with mortality post myocardial infarction could not be explained by controlling for socioeconomic status, results for physical health outcomes need to be replicated in larger, population-based samples [2,3,7,8]. Studies of living with pets and mortality in persons without prior acute myocardial infarction are lacking.

Possible mediators of the hypothesized association of companion animals with better human health have been suggested. Evidence is growing that dog or cat ownership is associated with better mental health, better coping with adverse or stressful life events, more physical activity, and possibly better social integration and support, all possibly leading to decreased chronic sympathoadrenal activation, improved immune function, and less chronic inflammation and physical health benefits [10–12]. Some researchers suggest more proximate affects of companionship and physical contact with a dog or cat on psycho-physiological pathways, though data are conflicting [10–12]. Health hazards of living with companion animals include infection, allergy and injury [13–15].

The present study sought to test the following hypotheses: (1) living with canine companion animals is associated with increased leisure time physical activity (LTPA); (2) living with companion animals is associated with reduced risk of death in subsequent years.

## 2. Experimental Section

### 2.1. Subjects

The Third National Health and Nutrition Examination Survey (NHANES III) was conducted in 1988–1994 on a nationwide multi-stage probability sample of 39,695 persons from the civilian, non-institutionalized population aged 2 months and over of the United States. Persons aged 60 and over, African Americans, and Mexican Americans were oversampled. Details of the plan, sampling, operation, response and institutional review board approval have been published as have procedures used to obtain informed consent and to maintain confidentiality of information obtained [16]. The personal interviews and physical and laboratory examinations of NHANES III subjects provided the baseline data for the study. This analysis was based on the public-use version of the NHANES III linked-mortality file with mortality data through 2000. Of 33,994 persons with baseline interview data, 13,944 were under age 17 and 26 lacked data for matching leaving 20,024 eligible for mortality follow-up. Of the 20,022 interviewed persons with mortality follow-up, 11,433 were aged 40 years and over, 11,394 of whom had valid data on companion animals and LTPA. Of these 3,187 died during the follow-up period. After excluding persons with missing data for any of the variables shown in Tables 1 and 2, 5,903 persons aged 40 and over with complete data remained for mortality analyses. The length of follow-up of survivors averaged 8.5 years.

### 2.2. Variables

During a home interview, interviewers asked questions concerning animals living in the household (exposure variable, Table 1), and collected demographic variables such as age, gender, race/ethnicity (non-Hispanic white, non-Hispanic black, Mexican American, others), and years of education completed [16]. Health status was assessed as self-reported general health, presence or absence of any history of major morbidity by physician diagnosis (heart attack, heart failure, stroke, medication for hypertension, diabetes, chronic bronchitis, emphysema, or non-skin cancer) and limitation of mobility (self-reported difficulty in climbing one flight of stairs or walking ¼ mile with survey physician impression of mobility used to impute missing data). Other variables collected are shown in Tables 1 and 2. To assess LTPA, participants were asked, “In the past month did you…?” (Yes/No). If yes, “In the past month, how often did you…?” (specify number of times), for the following: jogging or running, riding a bicycle or exercise bicycle, swimming, aerobic dancing, other dancing, calisthenics or floor exercises, gardening or yard work and weight lifting.” Open-ended questions assessed up to four other activities. Frequency of walking a mile or more was also asked. Persons responding “no times” to all of the above were classified “no LTPA.” Four frequency of activity groups were formed (0, 1–4, 5–7, and 8+ times/week) to divide active persons into similar-sized groups to facilitate analysis. Measurement of blood pressure, height, weight and serum analytes is described elsewhere [16].

To obtain the mortality outcome variables, a mortality linkage was done based upon the results from a probabilistic match of NHANES III participants with the National Death Index. The NHANES III linked mortality file includes deaths for adult participants occurring from the date of NHANES III interview in 1988–1994 through December 31, 2000. Information regarding the date of death and age of death was collected from matched death certificates. For details about NHANES III Linked Mortality Files see http://www.cdc.gov/nchs/r&d/nchs_datalinkage/nhanes_data_linkage_activities.htm.

### 2.3. Statistical Analysis

Detailed descriptive statistics and measures of association were computed using the SUDAAN system (Version 9.0, Research Triangle Institute, Research Triangle Park, NC), to take into account the complex survey design in producing variance estimates using Taylor series linearization for variance estimation [17]. Kaplan-Meier survival curves were computed using PROC KAPMEIER. Estimates of the risk of death derive from Cox proportional hazards regression models with time to event as the time scale computed using the SURVIVAL procedure in SUDAAN. Survivors were censored at the date of the end of mortality follow-up. In a hierarchical modeling strategy, three models were fit. Validity of the proportional hazards assumption was confirmed by inspection of un-weighted log negative log survival curves [18].

## 3. Results and Discussion

### 3.1. Results

Among persons aged 40 and over in bivariate analyses, cross-tabulation of living with companion animals (none, cat/other, dog) with frequency of LTPA (0, 1–4, 5–7, and 8+ times/week) was significant (p = 0.0003). Those living with a canine companion were most likely to be in the highest activity group (25%, 95% CL 21–31) and least likely to be in the no activity group (15%, 95% CL 13–18). The crude percentage of persons dying during the follow-up was lowest among those living with canine companions and highest among those with no animals in the household (p = 0.0002). Table 2 shows age-adjusted prevalence of selected characteristics by animals in the household. Companion animal frequency was associated with age, race/ethnicity, marital status, education, mobility limitation, religious attendance, systolic blood pressure and body mass index, but associations with activity and death were reduced. In linear regression models, the log frequency of moderate/vigorous LTPA was no longer significantly associated with living with animals after adjusting for age or multiple socio-demographic variables.

Age-specific Kaplan Meier survival curves showed the similar survival in those living with no companion animal, those living with a cat or other animal and those with living with a canine companion (log rank tests NS). In a bivariate regression model, persons living with a canine (HR 0.69, 95% CL 0.58–0.82, p = 0.0001) or a feline/other companion animal (HR 0.70 95%CL 0.57–0.85, p = 0.0005) had a lower risk of dying than those living with no companion animals. In models to test for selected interactions, no significant interaction was found for age, gender, ethnicity with living with companion animals.

After controlling for confounding by baseline age, gender and race/ethnicity, those living with companion animals had a slight but significantly increased risk of death (Table 3, Model I). Further controlling for socioeconomic variables and health status and activity level (Model II), revealed a similar pattern (Table 3). Finally, after controlling for healthy behaviors and other risk factors, this pattern persisted but was no longer statistically significant (Model III).

### 3.2. Discussion

This analysis of data from the NHANES III linked-mortality file, a nation-wide representative sample, is the one of the first studies to provide population-based data on the association of living with companion animals and survival. Living with companion animals was not independently associated with better survival despite its positive association with baseline physical activity and the putative psychosocial benefits reported in the literature [2–12].

Reasons for the lack of positive association of companion animals with survival may include the following. Living in the same household with a dog may be of little benefit if the person is not the one who walks and interacts with the dog. In fact the person may even have negative feelings about the animal. Further, pets can pose a health hazard to some people through infection, allergy and injury [13–15]. For example, a recent report indicates that animals in the household may increase the risk of falls among the elderly [15]. Thus, benefits of dog-related increased physical activity for some may have been cancelled by lack of benefit or even harm to spouses or others for whom no increased activity or other benefits accrued. Unfortunately no data were available in this survey to determine the relationship of the sample person with the animal(s) in their household or duration of animals’ presence, limiting interpretation of the data.

## 4. Conclusions

In a nationwide cohort of American adults, analyses demonstrated no relationship of living with companion animals and risk of death independent of confounders.

## Figures and Tables

**Table 1.** Exposure variable questionnaire items.

Do any pets live in this home?	1 Yes	2 No
What kind of pet lives here…?		
A dog?	1 Yes	2 No
A cat?	1 Yes	2 No
A bird?	1 Yes	2 No
A fish?	1 Yes	2 No
Any other pet?	1 Yes	2 No
Rodent	1 Yes	2 No
Rabbit	1 Yes	2 No
Reptile	1 Yes	2 No
Farm pet	1 Yes	2 No
Other	1 Yes	2 No

**Table 2.** Age-adjusted prevalence (%) of selected characteristics and living with companion animals in persons aged 40+y: NHANES III.

	Percentage				
	No pets	Other pet	Dog	Total	p
Aged 70–89 y	27	12	13	21	
Male	47	46	44	46	0.29
Mexican American	4	3	3	3	0.00
African American	13	6	5	10	
European American	76	87	87	81	
South region	36	35	28	34	0.03
Metropolitan residence	47	49	53	48	0.31
Married	62	73	64	66	0.00
Educ < 12 y	33	29	28	31	0.003
Fair-poor health	23	22	21	22	0.49
>=1 chronic illness	41	41	41	40	0.97
Mobility limitation	21	22	19	21	0.33
Religious attendance >= 52/y	44	36	35	41	0.00
Current smoking	22	28	22	24	0.01
Alcohol in past month	37	41	41	39	0.01
Low physical activity	79	82	83	81	0.77
No regular physician	24	21	24	23	0.12
Low social support	87	86	85	87	0.60
Systolic BP >=140mmHg	32	32	29	31	0.29
BMI >= 25 kg/m2	56	58	53	56	0.09
C-reative protein >3ng/L	36	34	34	35	0.53
Death	18	20	19	18	0.24
N	7,706	2,428	1,250	11,384	

**Table 3.** Adjusted hazards ratios (95% confidence intervals) of living with companion animals for mortality from all causes among persons aged 40 years and over in NHANES III.

Variable		Hazard ratio	95% CI	Hazard ratio	95% CI	Hazard ratio	95% CI

		Model	I	Model	II	Model	III
Companion animals	None	1.00		1.00		1.00	
	Cat/ other	1.24*	1.06–1.45	1.21*	1.05–1.39	1.11	0.93–1.32
	Dog	1.25*	1.07–1.45	1.21+	1.04–1.41	1.17	0.94–1.46
LTPA (times/wk)	0			1.00		1.00	
	1–4			0.60*	0.52–0.69	0.67*	0.56–0.80
	5–8			0.62*	0.51–0.76	0.64*	0.49–0.83
	> 8			0.54*	0.44–0.66	0.65*	0.52–0.81
Age	Yr	1.09*	1.09–1.10	1.08*	1.07–1.09	1.08*	1.07–1.09
Gender	Male	1.45*	1.32–1.58	1.63*	1.47–1.81	1.68*	1.47–1.91
Race/Ethnicity	AA	1.46*	1.30–1.64	1.15+	1.01–1.30	1.12	0.91–1.38
	MA	0.93	0.79–1.10	0.73*	0.61–0.88	0.84	0.66–1.07
Education	>= HS			0.96	0.86–1.07	1.04	0.89–1.21
South region	No			1.08	0.94–1.24	1.14	0.96–1.37
Metro area	Yes			1.07	0.92–1.23	1.07	0.89–1.28
Self–reported health	Good/excellent			0.59*	0.52–0.67	0.67*	0.56–0.79
Morbidity	No			0.59*	0.52–0.68	0.64*	0.54–0.77
Mobility	Not Limited			0.81*	0.71–0.92	0.64*	0.54–0.75
Systolic blood pressure, mmHg	Mm Hg					1.00+	1.00–1.01
Body mass index	Kg/m2					0.96*	0.95–0.98
Smoking	Current					1.84*	1.50–2.26
	Former					1.25*	1.06–1.48
Alcohol use	Yes					1.23	0.98–1.53
Reg. physician	No					0.97	0.82–1.16
Ln CRP						1.27*	1.18–1.37
LnHDL						1.00	0.99–1.00
Religious services	>=52/y					0.91	0.81–1.02

CI, confidence interval; CRP, C-reactive protein; AA, African American, MA, Mexican American, HS, high school, F/P fair-poor; Reg. regular care by personal physician;

* p <= 0.01,

† P < 0.05.
